# Iceland: an underestimated hub for the spread of high-pathogenicity avian influenza viruses in the North Atlantic

**DOI:** 10.1099/jgv.0.001985

**Published:** 2024-05-02

**Authors:** Ann Kathrin Ahrens, Stefán Ragnar Jónsson, Vilhjálmur Svansson, Brigitte Brugger, Martin Beer, Timm C. Harder, Anne Pohlmann

**Affiliations:** 1 Institute of Diagnostic Virology, Friedrich-Loeffler-Institut, Suedufer 10, 17493 Greifswald –Isle of Riems, Germany; 2 The Institute for Experimental Pathology at Keldur, University of Iceland, Reykjavík, Iceland; 3 The Icelandic Food and Veterinary Authority (MAST), Austurvegi 64, Selfossi, Iceland

**Keywords:** avian influenza, Iceland, wild birds

## Abstract

High-pathogenicity avian influenza viruses (HPAIVs) of the goose/Guangdong lineage are enzootically circulating in wild bird populations worldwide. This increases the risk of entry into poultry production and spill-over to mammalian species, including humans. Better understanding of the ecological and epizootiological networks of these viruses is essential to optimize mitigation measures. Based on full genome sequences of 26 HPAIV samples from Iceland, which were collected between spring and autumn 2022, as well as 1 sample from the 2023 summer period, we show that 3 different genotypes of HPAIV H5N1 clade 2.3.4.4b were circulating within the wild bird population in Iceland in 2022. Furthermore, in 2023 we observed a novel introduction of HPAIV H5N5 of the same clade to Iceland. The data support the role of Iceland as an utmost northwestern distribution area in Europe that might act also as a potential bridging point for intercontinental spread of HPAIV across the North Atlantic.

## Data Summary

All associated information and source data are available on Zenodo under https://doi.org/10.5281/zenodo.10497319. All analysed sequences are available in the EpiFlu^TM^ database. We acknowledge the originating and submitting laboratories for sharing their sequence data in file https://zenodo.org/records/10497319/files/03_ICE2022_data_source_acknowledgment_table. csv?download=1.

## Introduction

Influenza A viruses, members of the family *Orthomyxoviridae*, have an eightfold-segmented single-stranded RNA genome that allows for reassortment with other influenza A viruses infecting the same host cell [1,2]. This process, together with an inaccurate viral replication machinery, fuels the remarkable genetic variability of these viruses [3,4]. An essential step in virus maturation, the endoproteolytic cleavage of the HA0 precursor protein, is catalysed by host cell-derived proteases [5]. The motif of the cleavage site determines the access of proteases suitable for processing. For avian influenza viruses, this has a direct impact on the pathotype of the virus [6]. Wild aquatic birds of the orders Anseriformes and Charadriiformes are considered the natural reservoir of AIV and are capable of transmitting AIV to other wild and domestic birds and, more rarely, even to mammals [7,8]. Replication of high-pathogenicity avian influenza virus (HPAIV) in poultry flocks leads to high mortality, especially in galliform poultry, within a few days. In addition, infected poultry flocks may serve as temporary amplification sites of HPAIV and through spill-back transmission may introduce the virus into local wild bird populations [9]. Depending on the virus strain and host species, different disease outcomes, ranging from asymptomatic to highly lethal, can be observed in wild birds [8].

In the mid-1990s, in Southeast Asia, an HPAIV strain designated A/goose/Guangdong/1/1996 (H5N1), abbreviated to gs/Gd, emerged in domestic poultry in PR China [10]. Continuing failure to contain the strain enabled its further spread regionally as well transcontinentally. Progressive reassortment of this strain with AIV of low pathogenicity (LP) in poultry and in wild birds gave way to a plethora of emerging gs/Gd HP genotypes. Other selective forces shaped up to 10 major, and an ever growing number of subordinated, phylogenetically defined clades of its haemagglutinin (H5) [10]. Viruses assigned to clade 2.3.4.4 occurred in 2013 in PR China for the first time [11]. The lineage split into further eight subclades (a–h) of which ‘c’ (in 2014) and ‘b’ (since 2016) occurred in Europe [10]. Evidence has accumulated to prove that HPAIVs of these clades can be transferred over long distances by wild birds along their migration pathways [12,13]. This has sparked a currently worldwide spread of clade 2.3.4.4b, sparing, so far, only Australia and Oceania [13,14]. The Americas were reached at least twice via the Bering Strait in 2014 but also by transmission across the (northern) Atlantic in 2021 when the first HPAIV 2.3.4.4b were detected in wild birds at the easternmost tip of Newfoundland [12]. A linkage of these cases to an European origin was immediately evident phylogenetically. Additional data revealed Iceland, located between Scotland and Canada in the North Atlantic, as a putative ‘stepping stone’ for this clade on its way to North America [15].

Here we have further phylogenetically analysed HPAIV H5 of the gs/Gd lineage detected in Iceland since 2021. The data show continuous new introductions but only from east to west, as no gene segments of American origin have been detected so far.

## Methods and Materials

### Sampling and initial molecular testing

A total of 165 samples for the year 2022 and 64 samples for 2023 from wild birds has been analysed by real time RT-PCR (RT-qPCR) for the presence of influenza A virus-specific RNA [16,17]. Positive samples were further screened by RT-qPCR for sub- and pathotype characteristics as described elsewhere [18]. Sample characteristics such as species, geographical origin on Iceland, matrix and tissue identity are summarized in Table S1, available in the online version of this article. Samples were collected between 15 April 2022 and 16 October 2022 and between 23 March 2023 and 31 October 2023. The Icelandic avian samples originate from carcasses; all sampling was with permission from the Icelandic Food and Veterinary Authority (MAST).

Initially all samples were RNA-extracted and examined in a generic real-time RT-PCR (RT-qPCR) format targeting a conserved region of the M gene segment; this and all further RT-qPCRs used also for sub- and pathotyping were according to previously published methods [18].

### MinION-based sequencing

MinION-based sequencing was restricted to avian influenza-positive samples with *C*_q_ values <28 due to quality issues as described before [19,20]. Briefly, the RNA was transcribed into DNA using the Superscript III One-Step and Platinum *Taq* kit (#12 574 026, Thermo Fisher Scientific, USA) with influenza-specific primers (Pan-IVA-1F: TCCCAGTCACGACGTCGTAGCGAAAGCAGG; Pan-IVA-1R: GGAAACAGCTATGACCATGAGTAGAAACAAGG), each of which bind at the conserved 3′ or 5′ end of all influenza RNA segments. DNA amplificates were purified with Agencourt AMPure XP magnetic beads (#A63881, Beckmann Coulter, Krefeld, Germany) using DNA LoBind tubes (#0030108051, Eppendorf, Wesseling-Berzdorf, Germany). Approximately 400 ng of DNA was used for sequencing by a transposase-based library preparation approach with Rapid Barcoding (SQK-RBK004, Oxford Nanopore Technologies, Oxford, UK) and a Flow Cell (R9.4.1) on a MinION Mk1B device with MinKNOW Software Core (v5.4.3). Live high-accuracy base calling of the raw data with Guppy (v6.4.4, Oxford Nanopore Technologies) was followed by demultiplexing, a quality check and a trimming step to remove low-quality, primer and short (<20 bp) sequences. The generated data were saved in FASTQ and POD5 data formats. The bioinformatic software suite Geneious Prime (Biomatters, Version 2021.0.1) was used for analysis. The sequences were trimmed to remove the primer sequences. Consensus sequences were obtained with an iterative map-to-reference approach with Minimap2 (v2.17). Reference genomes from a curated collection of all HA and NA subtypes alongside an assortment of internal gene sequences were chosen to cover all potentially circulating viral strains. Polishing of the final genome sequences and annotation was done manually after consensus generation (threshold matching 60 % of bases of total adjusted quality). Further methodological details and sequence accession information are summarized at https://doi.org/10.5281/zenodo.10497319.

### Phylogenetic analysis

Segment-specific and concatenated whole-genome multiple alignments were generated using MAFFT (v7.450) [21] and subsequent maximum-likelihood (ML) trees were calculated with RAxML (v8.2.11) [22] utilizing a GTR GAMMA model with rapid bootstrapping and searching for the best scoring ML tree supported with 1000 bootstrap replicates or alternatively with FastTree (v2.1.11) [23]. Subsets of closely related genomes were extracted and used for further ML phylogenetic analyses. Time-scaled trees of concatenated sequences of the different genotypes were calculated with the beast (v1.10.4) software package using a GTR GAMMA substitution model, an uncorrelated relaxed clock with a lognormal distribution and coalescent constant population tree models [24]. Chain lengths were set to 50 million iterations and convergence checked via Tracer (v1.7.1). Time-scaled summary maximum clade credibility (MCC) trees with 10 % post-burn-in posterior were created using TreeAnnotator (v1.10.4) and visualized with FigTree (V1.4.4). The MCC trees show 95 % highest posterior density (HPD) confidence intervals at each node and posterior confidence values as branch support. Spatio-temporal spread was inferred on MCC trees with country traits using SPREAD (v1.0.7) and visualized with QGIS (v3.24.3, QGIS.org) [24].

## Results and discussion

**Fig. 1.**
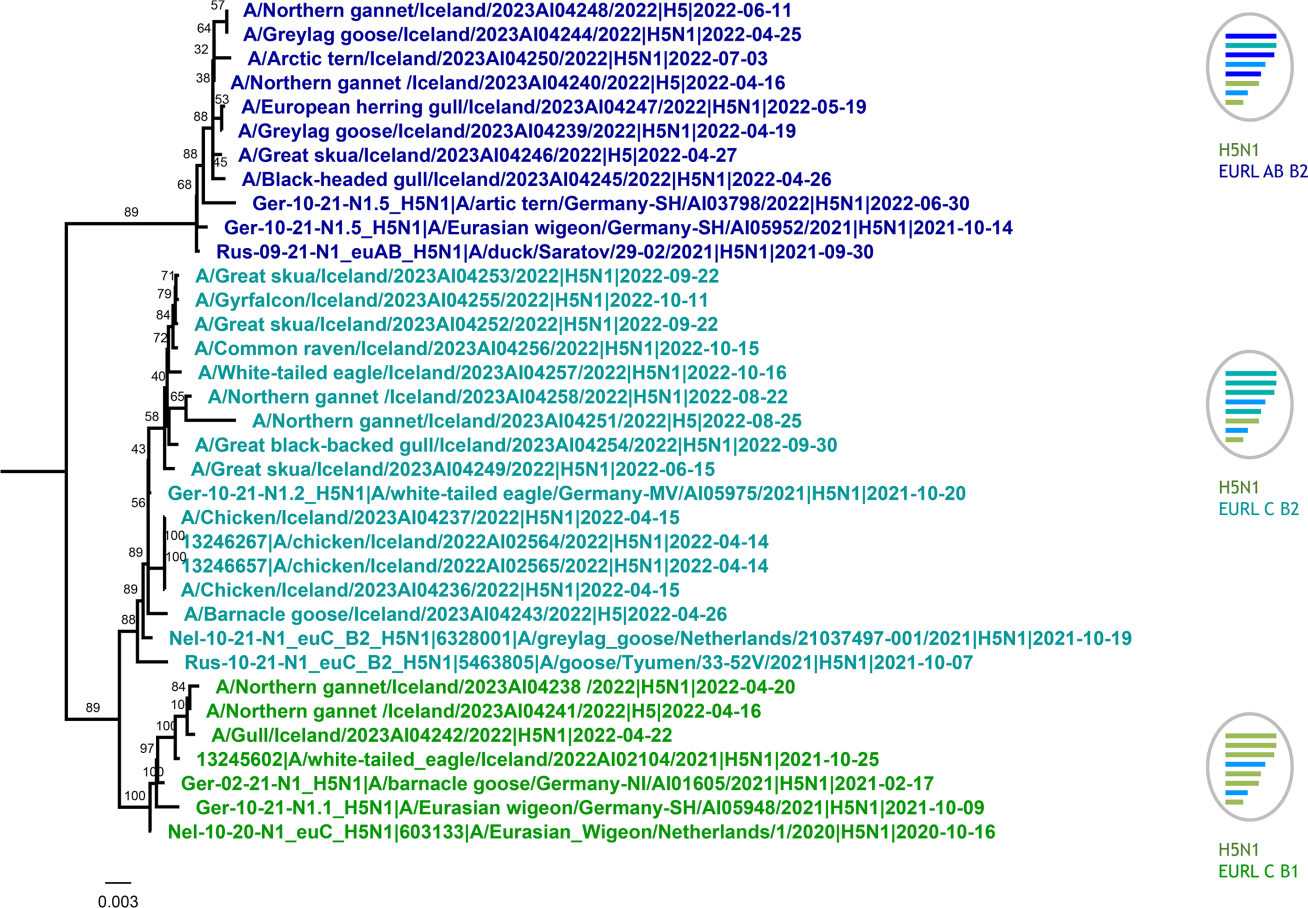
Phylogenetic analysis of sequences from Iceland collected 2021–2022 and assignment of European genotype specifications to HPAIV H5N1 samples derived from wild birds in Iceland. Concatenated genomes were aligned using MAFFT and trees were calculated with RAxML. A selection of reference sequences representing different genotypes described in Europe were used for comparison. Colours represent focus genotypes. Green, B1; aqua, B2 euC; blue, B2 euAB.

Of the 165 samples collected in 2022, 45 samples (27.4 %) tested positive for IAV. For the collection period of 2023, three samples of a total number of seven tested positive. All samples were tested by RT-qPCR for subtype H5 and a total of 37 samples out of the 45 samples from 2022 and 6 out of the 7 samples from 2023 harboured an H5 virus with a multibasic HA cleavage site, i.e. were of a high pathogenicity genotype as defined by a pathotype-specific RT-qPCR [18]. The N1-specific RT-qPCR detected 36 samples for 2022 that were positive, while 4 samples for 2023 were positive for N5. Metadata information and detailed test results are shown in Table S1. For MinION sequencing, 26 samples (2022 and 2023) were selected and 11 complete genomes and 6 incomplete genomes in which individual segments or parts thereof are missing were generated; 9 samples yielded partial genome fragments only. Out of the three samples from 2023 only one sample yielded a full genome sequence. Sequence analysis confirmed the PCR-based subtyping and pathogenicity characterization. Phylogenetic analysis showed that the HA sequences all clustered with gs/Gd-derived viruses of the 2.3.4.4b clade and full genotypes could be determined for 16 samples according to the methodology described [24]. Three different genotypes were found that have been previously described in wild birds on the European mainland: European (eu) genotype C was present in 11 samples, which can be further differentiated into subgroups B1 (*n*=1) and B2 (*n*=10) according to distinct H5 sequences within clade 2.3.4.4b as described elsewhere [25]. Detection of genotype euC-B2 was scattered across Iceland, whereas euC-B1 was restricted to a location in the west of Iceland. In addition, five samples were assigned to genotype euAB with an HA that assorted with the B2 lineage. The H5N5-positive sample collected in 2023 was characterized as genotype euI. The genome constellations are depicted in Fig. 1.

The distinct genotype classification also became evident when concatenated genome segments were put to phylogenetic analyses (Fig. 1). Genotype euC-B1 (green colour in Fig. 1) has been present in Europe since October 2020. The type strain of this genotype is A/Eurasian Wigeon/Netherlands/1/2020. Genotype euC-B2 (aqua) was present in Europe from November 2020 (type strain, A/Eurasian wigeon/Italy/20VIR7301-206/2020) and euAB-B2 (blue) was present in Europe/Russia from September 2021 onwards (type strain, A/duck/Saratov/29-02/2021). The genotypes share the same HA, NA, MP and NS segments, but differ in their polymerase (PB2, PB1, PA) and nucleoprotein (NP) encoding segments.

**Fig. 2.**
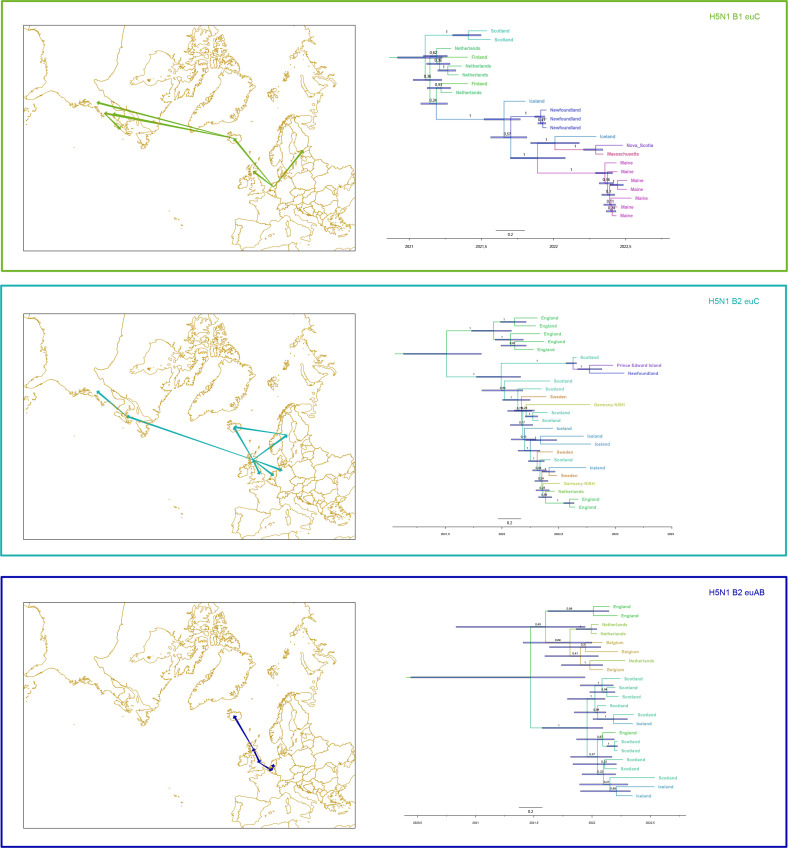
Phylogeographical analyses of sequences from Iceland collected 2021–2022. Time-scaled MCC trees were inferred using beast, with country as discrete trait. Spatial distribution visualized using SPREAD and QGIS. Colours represent focus genotypes. Green, B1 euC; aqua, B2; blue, B2 euAB.

Phylogeographical analyses based on time-scaled MCC trees and spatial spreading patterns are presented in Fig. 2. It should be noted that there is a marked imbalance of publicly available sequence entries from different geographical sites; we are aware of the influence of this skewness of data and the compromised reliability of phylogeographical estimates. According to these data, an incursion of genotype euC-B1 from Europe into Iceland occurred in early 2021 (time of the most recent common ancestor, MRCA [26]: ~2021.19); interestingly, at least two onward transmissions into North America later in 2021 (MRCA ~2021.66, ~2021.7) were associated with this genotype. The first cases of these viruses on the North American continent stem from late 2021 and are located at the most northwestern spot of Newfoundland [12,27]. Based on our distribution modelling, H5N1 euC-B1 was first detected in the Netherlands and, assuming that this was the starting point for further spread, the genotype spread in northeastern as well northwestern directions to Finland as well as to the UK and Iceland. From Iceland, the genotype made the leap across the Atlantic Ocean to the east coast of North America. Incursions into Iceland of genotype euC-B2 viruses likewise had their origin in the British Isles and could have been transferred in the first months of 2022 (MRCA ~2022.18). Viruses of this genotype also spread backwards from the British Isles to European mainland in summer 2022, mediated by colony-breeding seabirds, mainly gannets [15]. Based on phylogeographical analysis (inferred from sequences Different location and MRCA) it could be assumed that there was a second incursion of this genotype into Iceland, probably from Scandinavia (Sweden) during the summer of 2022 (MRCA ~2022.42). In addition, this genotype was also transferred to North America by independent spread, probably directly from the British Isles in late 2022 (MRCA ~2022.63). The incursion of genotype euAB-B2 (blue) into Iceland dated to early 2022 (MRCA ~2022.18). This genotype has not been detected on the North American continent so far. The H5N1 euAB-B2 genotype can be roughly distinguished into a northern and a southern European phylogenetic lineage. The Nordic lineage has been found in Iceland, England and Scotland and other European countries, while the southern clade was detected in the Netherlands and Belgium but also in England.

H5N5 is not a novel subtype, as it was circulating in different European countries and also for different periods of time in 2023 in Norway (Svalbard), Iceland, the Faroe Islands and the UK [28,30]. In addition, Canada reported an H5N5-positive fox in April 2023 [31].

In conclusion, our data confirm that Iceland is involved in the circulation of clade 2.3.4.4b HPAI H5N1 viruses both as a sink for viruses from continental European and as a source for North America. It is therefore reasonable to assume that Iceland could act as a gateway for avian influenza in the transatlantic transmission pathway. However, to date there is no evidence of west-to-east transmission. Nevertheless, Iceland remains an important sampling area for the detection of novel avian influenza incursions. Iceland also remains a focal point for ongoing monitoring with regard to the potential impact of the HPAI epidemic in seabirds on biodiversity and the protection of endangered species.

## Software

For the sequence analysis Geneious Prime (version 2021.0.1), Biomatters, Inc., Auckland, New Zealand was used.

## supplementary material

10.1099/jgv.0.001985 Table S1.

## Abbreviations

AIV: avian influenza virus
gs/Gd: Goose/Guandong
HA: haemagglutining protein
HP: high-pathogenicity
HPD: highest posterior density
IAV: influenza A virus
LP: low-pathogenicity
MCC: maximum clade credibility
MRCA: most recent common ancestor
RT-qPCR: real time RT-PCR
