# Supplementary material for: Iceland: an underestimated hub for the spread of high-pathogenicity avian influenza viruses in the North Atlantic
Source: J Gen Virol. 2024 May 2;105(5):001985. doi: 10.1099/jgv.0.001985 (PMC11170123; doi:10.1099/jgv.0.001985)
Supplement: Table S1. [file jgv-105-01985-s001.pdf]

Host common name	Swab sample	WB	FLI-ID	Collection date	Location	Ct	Sequence Name	Full genome	Subtype	Genotype	Genotype EURL
Chicken	pharynx/cloaca	AF01	2023AI04236	2022-04-15	S-ICE	17.2	A/Chicken/Iceland/2023AI04236/2022|H5N1|2022-04-15	incomplete	H5N1	Ger-10-21-N1.2	C
Chicken	pharynx/cloaca	AF02	2023AI04237	2022-04-15	S-ICE	11.8	A/Chicken/Iceland/2023AI04237/2022|H5N1|2022-04-15	complete	H5N1	Ger-10-21-N1.2	C
Northern gannet	pharynx	WB-028-1	2023AI04238	2022-04-20	SW-ICE	19.8	A/Northern gannet/Iceland/2023AI04238 /2022|H5N1|2022-04-20	partial	H5N1		
Greylag goose	pharynx	WB-031-1	2023AI04239	2022-04-19	N-ICE	14.9	A/Greylag goose/Iceland/2023AI04239/2022	complete		Ger-10-21-N1.5	AB
Northern gannet	pharynx	WB-033-1	2023AI04240	2022-04-16	W-ICE	22.5	A/Northern gannet /Iceland/2023AI04240/2022|H5|2022-04-16	partial	H5Nx		
Northern gannet	pharynx	WB-034-1	2023AI04241	2022-04-16	W-ICE	15.9	A/Northern gannet /Iceland/2023AI04241/2022|H5|2022-04-16	partial	H5Nx		
Great black-backed gull	pharynx	WB-041-1	2023AI04242	2022-04-22	W-ICE	14.0	A/Gull/Iceland/2023AI04242/2022|H5N1|2022-04-22	complete	H5N1	Ger-10-21-N1.1	C
Barnacle goose	pharynx	WB-046-1	2023AI04243	2022-04-26	NW-ICE	21.1	A/Barnacle goose/Iceland/2023AI04243/2022|H5|2022-04-26	incomplete	H5Nx	Ger-10-21-N1.2	C
Greylag goose	pharynx	WB-049-2	2023AI04244	2022-04-25	N-ICE	17.4	A/Greylag goose/Iceland/2023AI04244/2022|H5N1|2022-04-25	complete	H5N1	Ger-10-21-N1.5	AB
Black-headed gull	pharynx	WB-059-1	2023AI04245	2022-04-26	N-ICE	22.3	A/Black-headed gull/Iceland/2023AI04245/2022|H5N1|2022-04-26	complete	H5N1	Ger-10-21-N1.5	AB
Great skua	pharynx	WB-061-1	2023AI04246	2022-04-27	N-ICE	23.1	A/Great skua/Iceland/2023AI04246/2022|H5|2022-04-27	partial	H5Nx		
European herring gull	pharynx	WB-092-1	2023AI04247	2022-05-19	N-ICE	17.8	A/European herring gull/Iceland/2023AI04247/2022|H5N1|2022-05-19	incomplete	H5N1	Ger-10-21-N1.5	AB
Northern gannet	pharynx/cloaca	WB-099	2023AI04248	2022-06-11	SW-ICE	30.8	A/Northern gannet/Iceland/2023AI04248/2022|H5|2022-06-11	partial	H5Nx		
Great skua	pharynx	WB-101-1	2023AI04249	2022-06-15	E-ICE	19.3	A/Great skua/Iceland/2023AI04249/2022|H5N1|2022-06-15	complete	H5N1	Ger-10-21-N1.2	C
Arctic tern	pharynx/cloaca	WB-111	2023AI04250	2022-07-03	SE-ICE	16.8	A/Arctic tern/Iceland/2023AI04250/2022|H5N1|2022-07-03	complete	H5N1	Ger-10-21-N1.5	AB
Northern gannet	pharynx/cloaca	WB-122	2023AI04251	2022-08-25	N-ICE	32.3	A/Northern gannet/Iceland/2023AI04251/2022|H5|2022-08-25	partial	H5Nx		
Great skua	pharynx	WB-125	2023AI04252	2022-09-22	SW-ICE	15.3	A/Great skua/Iceland/2023AI04252/2022|H5N1|2022-09-22	incomplete	H5N1	Ger-10-21-N1.2	C
Great skua	pharynx	WB-126	2023AI04253	2022-09-22	SW-ICE	17.1	A/Great skua/Iceland/2023AI04253/2022|H5N1|2022-09-22	complete	H5N1	Ger-10-21-N1.2	C
Great black-backed gull	3x(pharynx/cloaca)	WB-129A	2023AI04254	2022-09-30	SW-ICE	18.0	A/Great black-backed gull/Iceland/2023AI04254/2022|H5N1|2022-09-30	complete	H5N1	Ger-10-21-N1.2	C
Gyrfalcon	pharynx	WB-135A	2023AI04255	2022-10-11	SE-ICE	21.5	A/Gyrfalcon/Iceland/2023AI04255/2022|H5N1|2022-10-11	incomplete	H5N1	Ger-10-21-N1.2	C
Common raven	pharynx/cloaca	WB-136	2023AI04256	2022-10-15	S-ICE	23.4	A/Common raven/Iceland/2023AI04256/2022|H5N1|2022-10-15	incomplete	H5N1	Ger-10-21-N1.2	C
White-tailed eagle	pharynx/cloaca	WB-138	2023AI04257	2022-10-16	W-ICE	20.2	A/White-tailed eagle/Iceland/2023AI04257/2022|H5N1|2022-10-16	complete	H5N1	Ger-10-21-N1.2	C
Northern gannet	2x(pharynx)/1x(cloaca)	WB-142	2023AI04258	2022-08-22	E-ICE	27.6	A/Northern gannet /Iceland/2023AI04258/2022|H5N1|2022-08-22	partial	H5N1		
Mallard	cloaca	WB 05	2023AI08589	2023-03-23	SW-ISL	31.97		partial			
White tailed eagle	n.i.	WB 045_3	2023AI08560	2023-09-23	W-ISL	24.08		partial			
Common eider	cloaca	WB 047	2023AI08561	2023-09-25	N-ISL	21.37		complete	H5N5		I

Table S1: Metadata of the analyzed samples.

The table presents a short overview on the main important information for samples and analysis results. Abbreviations: n.i. – no information available; ICE – Iceland; E – East; N – North; W – West; S – South (in combination: S-ICE means South Iceland), WB – Icelandic identification number; * - Ct value obtained by influenza A virus-generic M-specific RT-qPCR.
